# Supplementary material for: Upconversion amplification through dielectric superlensing modulation
Source: Nat Commun. 2019 Mar 27;10:1391. doi: 10.1038/s41467-019-09345-0 (PMC6437158; doi:10.1038/s41467-019-09345-0)
Supplement: Supplementary file 1 — Supplementary Information [file 41467_2019_9345_MOESM1_ESM.pdf]

Supplementary for

Upconversion Amplification through Dielectric Superlensing Modulation

Liangliang Liang[1], Daniel B. L. Teh[2,7], Ngoc-Duy Dinh[3], Weiqiang Chen[4], Qiushui Chen[1], Yiming Wu[1], Srikanta Chowdhury[5,6], Akihiro Yamanaka[5,6], Tze Chien Sum[4], Chia-Hung Chen[7,8], Nitish V. Thakor[3,7], Angelo H. All[9] , and Xiaogang Liu[1,7,10*]

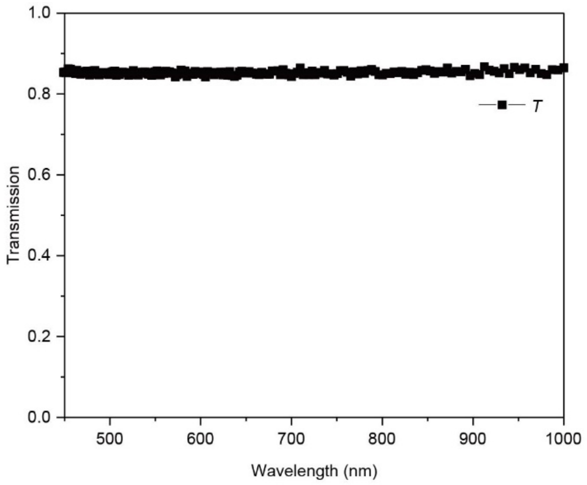

Supplementary Figure 1. FDTD simulation result for transmission (*T*) spectrum of a close-packed dielectric microbead monolayer. The simulation result indicates that the dielectric microbead film does not show any photonic crystal effect in the visible range.

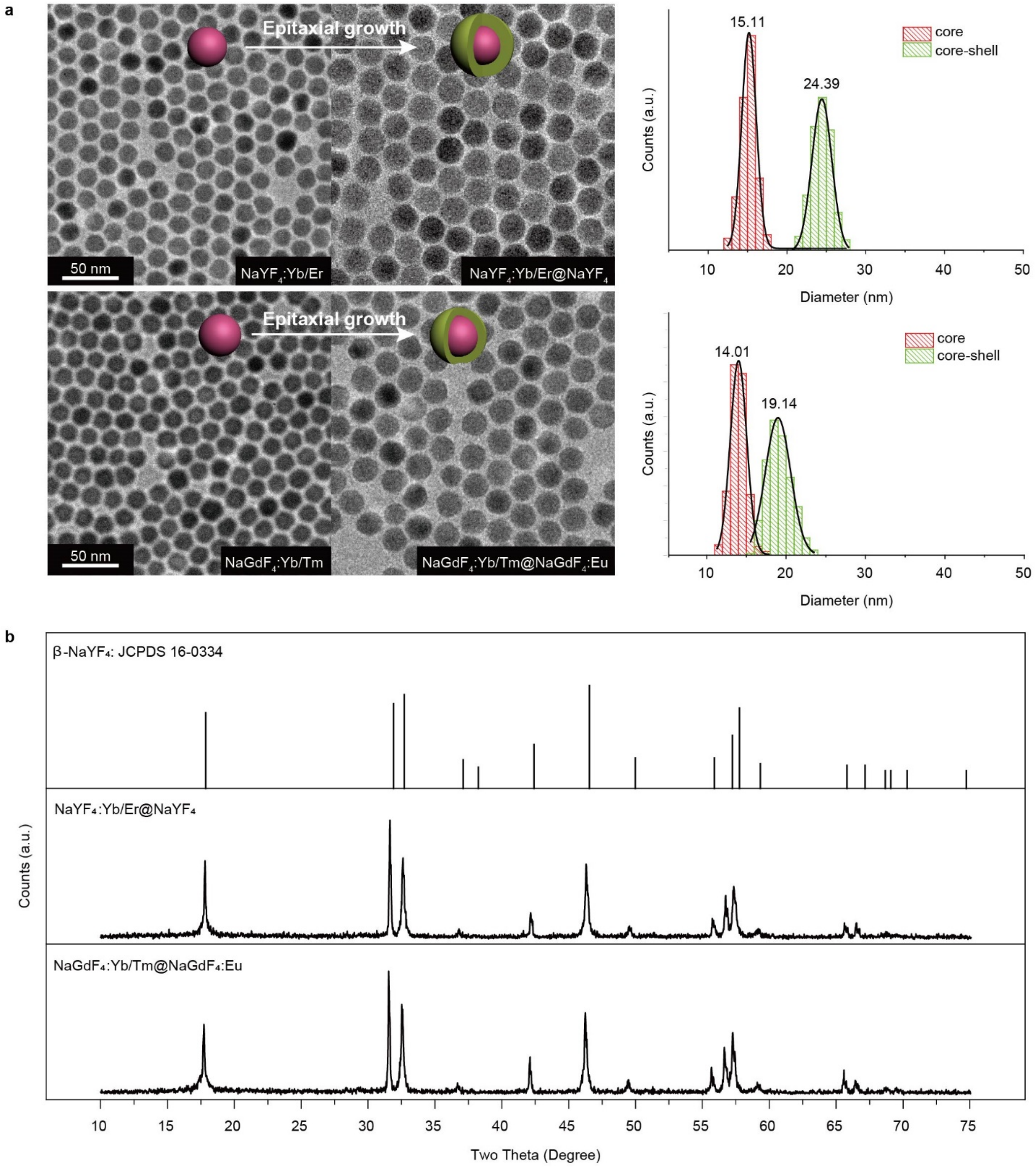

Supplementary Figure 2. TEM and XRD characterizations of the as-prepared hexagonal upconversion nanocrystals. a, TEM images and the corresponding size distribution of core and core-shell upconversion nanocrystals. **b,** X-ray diffraction patterns for powder upconversion nanocrystals. All peaks are indexed in accordance with hexagonal-phase $NaYF_4$ structure (Joint Committee on Powder Diffraction Standards file number 16-0334).

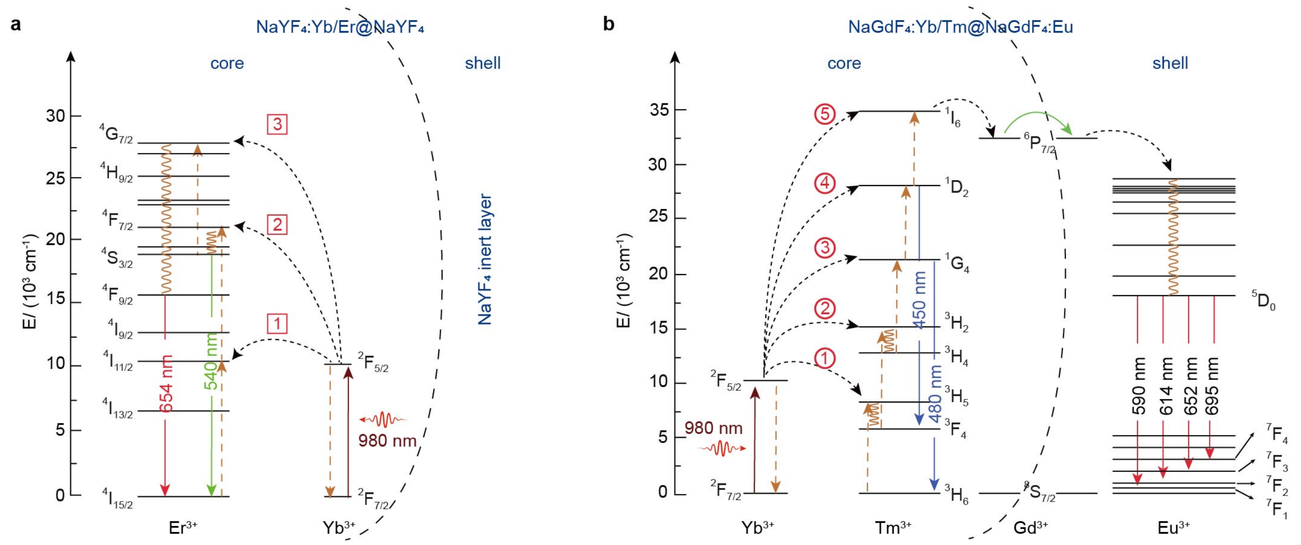

Supplementary Figure 3. Proposed energy transfer upconversion mechanisms of upconversion nanocrystals. Upconversion population and emission processes of (**a**) Er^{3+} and (**b**) Tm^{3+}/Eu^{3+} activated core-shell upconversion nanocrystals at 980 nm laser excitation. The $^{2S+1}L_J$ notations used to label the f levels refer to spin (S), orbital (L), and angular (J) momentum quantum numbers, respectively. An inert shell layer of $NaYF_4$ was added to mitigate surface quenching of luminescence.

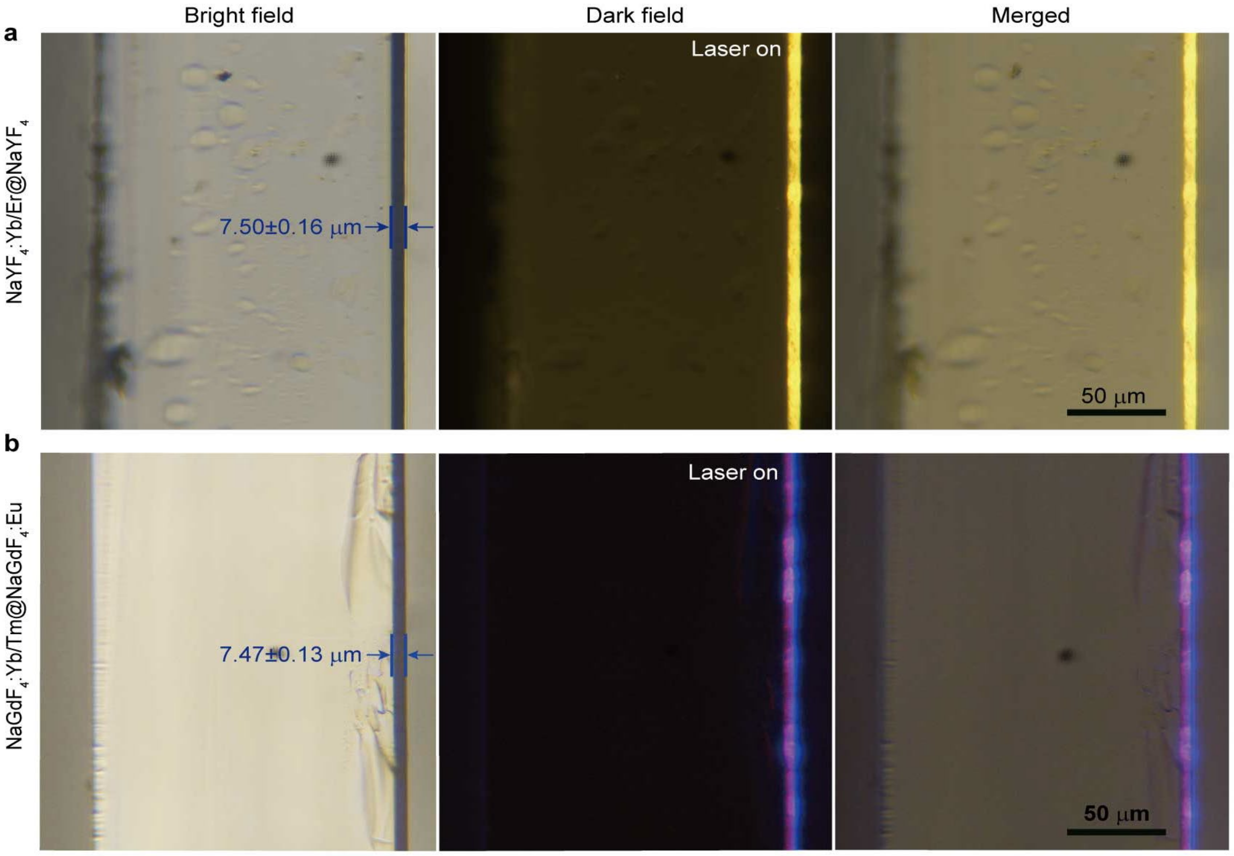

Supplementary Figure 4. Thickness characterization of UCNP-embedded PDMS layers (cross-sectional view) by fluorescence microscopy. Bright field and upconversion emission images of PDMS films containing (**a**) $NaYF_4$:Yb/Er@$NaYF_4$ and (**b**) $NaGdF_4$:Yb/Tm@$NaGdF_4$:Eu nanoparticles.

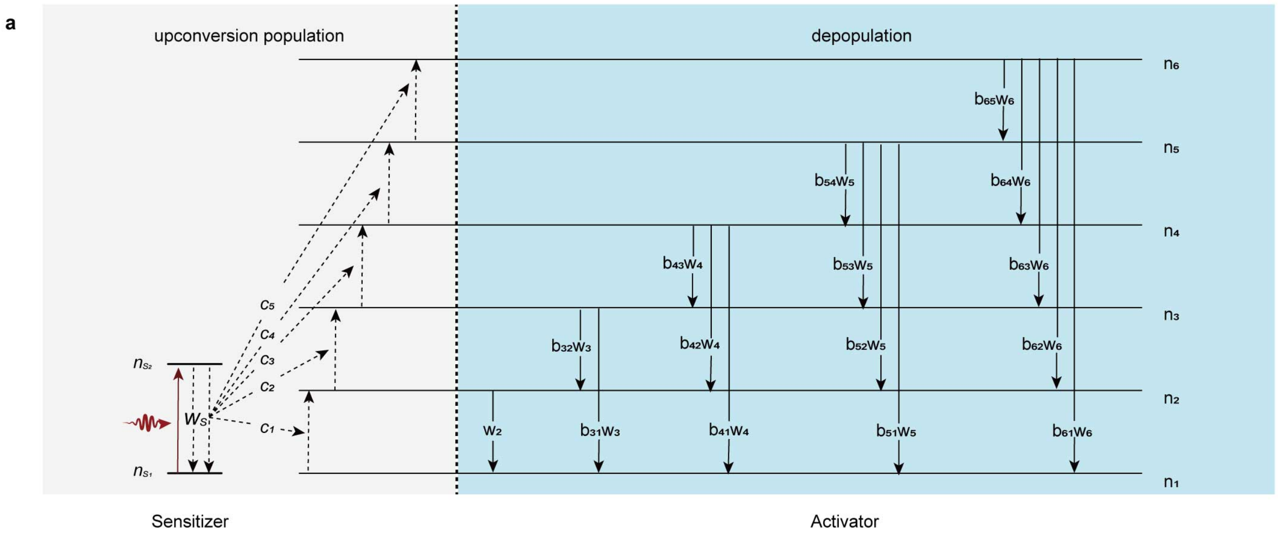

b

Kinetic Parameters

Intrinsic decay constants (s^{-1})

W_S	W_2	W_3	W_4	W_5	W_6
1000	500	1000	1500	2000	2500

upconversion coefficients (cm^3s^{-1})

C_1	C_2	C_3	C_4	C_5
1×10^{-17}	1×10^{-17}	1×10^{-17}	1×10^{-17}	1×10^{-17}

branching ratios

b_{31}	b_{32}	b_{41}	b_{42}	b_{43}	b_{51}	b_{52}	b_{53}	b_{54}	b_{61}	b_{62}	b_{63}	b_{64}	b_{65}
0.3	0.7	0.2	0.3	0.5	0.1	0.2	0.3	0.4	0.05	0.1	0.15	0.25	0.45

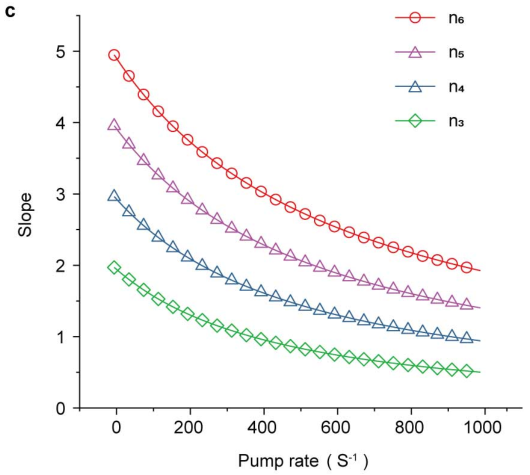

Supplementary Figure 5. Numerical modeling of general sensitizer-activator energy transfer upconversion system. a, Simplified six-level energy diagram of an energy transfer upconversion system containing sensitizers and activators. **b,** Proposed rate parameters for the general sensitizer-activator upconversion system simulation. **c,** Numerically simulated evolutions of the sensitivity of upconversion luminescence intensity as a function of pump rate.

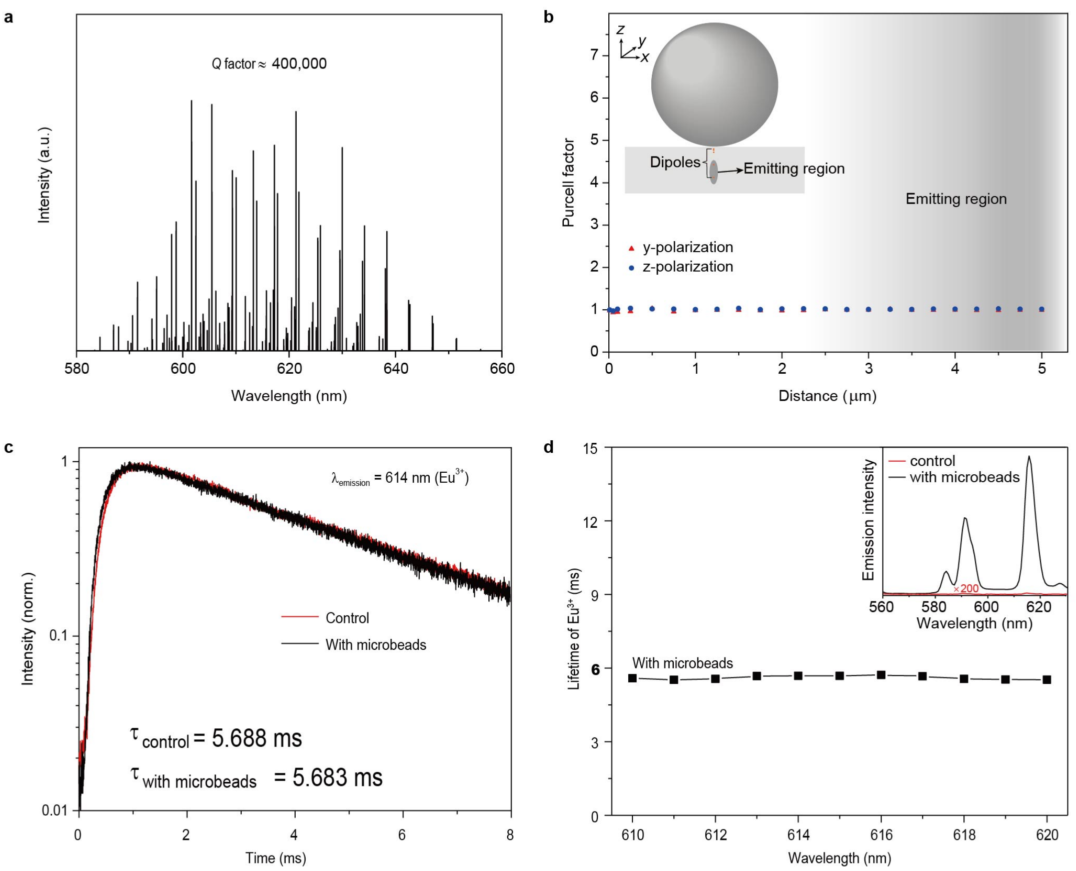

Supplementary Figure 6. a, Simulated resonance spectra of a microbead with a dipole source attached on its surface. **b,** Purcell factors of a dipole source placed at different locations (0.01 to 5 μm) with respect to the surface of the microbead. **c,** Upconversion luminescence lifetimes of Eu^{3+} at 614 nm (emission peak) with and without the microbead. **d,** Upconversion luminescence lifetimes of Eu^{3+} across the whole emission band from 610 to 620 nm with 1 nm-step for each measurement. Inset: emission spectra of Eu^{3+}-mediated photon upconversion with and without the microbead.

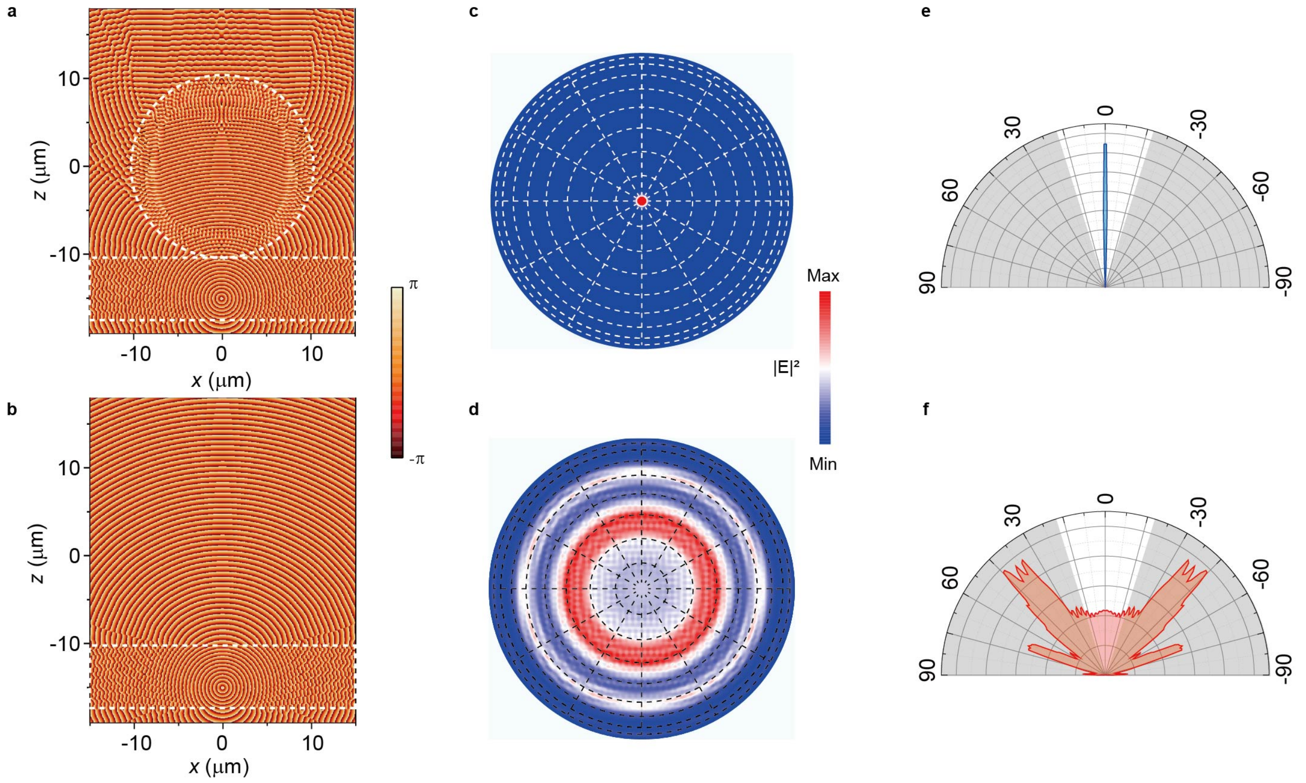

Supplementary Figure 7. FDTD simulation of wavefront modulation and far-field emission light projection through a dielectric microbead. a and **b,** The emitting light propagation phase map from a point light source after passing through a PDMS film with (**a**) and without (**b**) a dielectric microbead on the top, respectively. It is should be noted that the dielectric microbead can convert the spherical wave of a dipole emission to a quasi-plane wave within a very short propagating distance (~ 20 μm). **c** and **d,** Upconversion luminescence far-field projection with (**c**) and without (**d**) the microbead on the top of the upconverting film. **e** and **f** are the corresponding emission pattern extracted from **c** and **d**. Here, a dipole source was used for point light source simulation, and the emission photons of 600 nm was chosen for light collection ability evaluation.

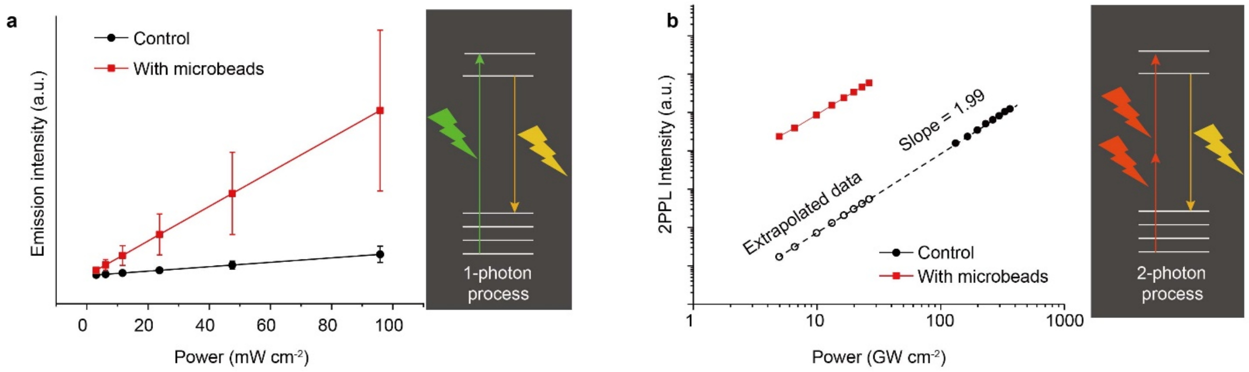

Supplementary Figure 8. Investigation of microbead-based fluorescence enhancement for Stokes and two-photon absorption photoluminescence of Rhodamine B dye. a, Linear optical fluorescence (one-photon) of Rhodamine B with and without the superlensing layer. Error bars represent ± 1 s.d. with n = 3 each. **b,** Simultaneous two-photon absorption fluorescence of Rhodamine B recorded before and after adding a superlensing layer. Insets in **a** and **b** illustrate the linear and two-photon absorption fluorescence processes, respectively. Note that for measurements without the microbead amplification, the simultaneous two-photon photoluminescence of Rhodamine B under low fluence was obtained by extrapolation that obtained under higher fluence, as the measured intensity under low excitation fluence could be extremely low and may lead to large errors of measurement.

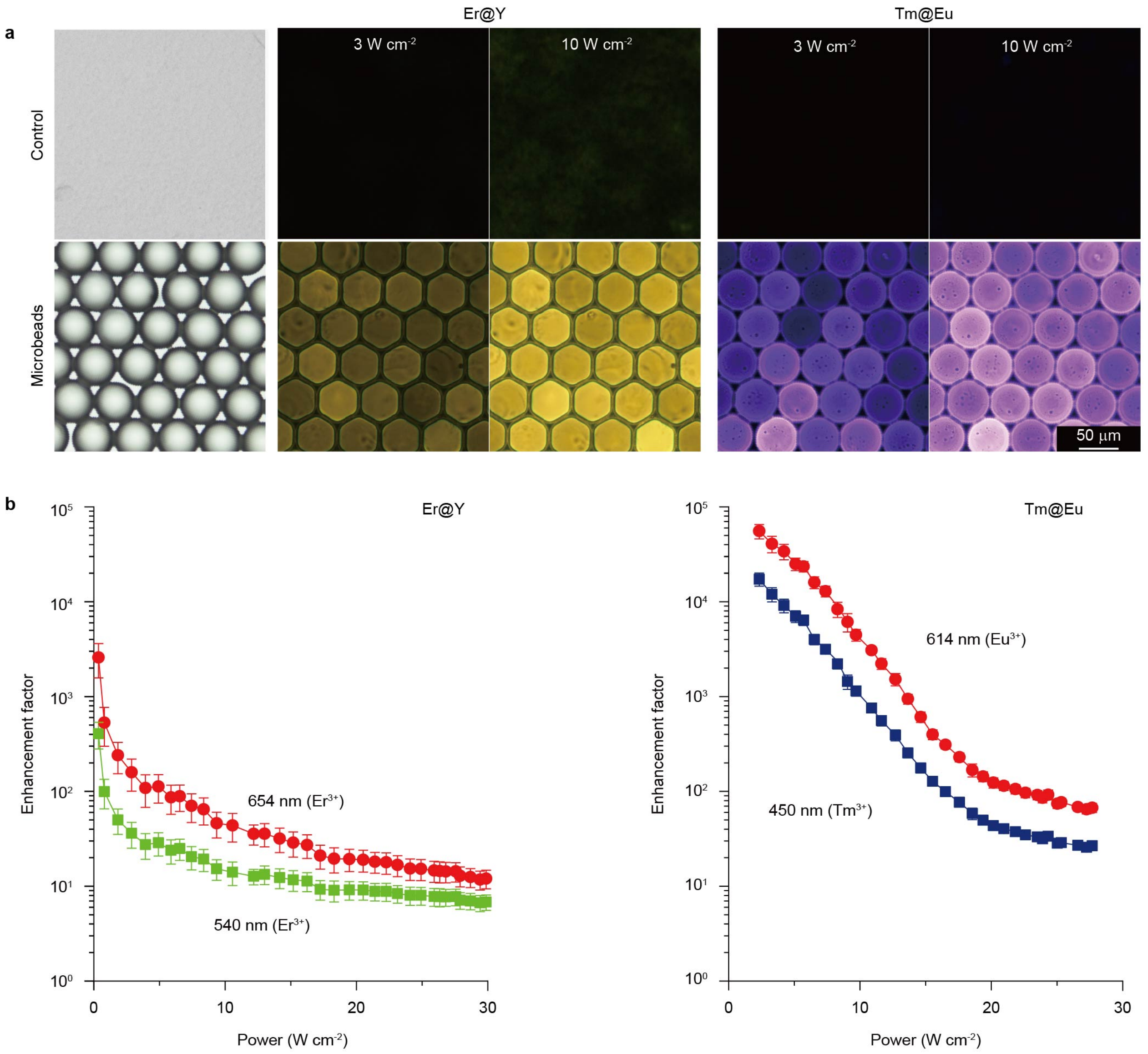

Supplementary Figure 9. Upconversion luminescence amplification features of poly (ethylene glycol) diacrylate microbeads with diameters around 50 μm. a, Comparison of the upconversion images obtained with and without the microbead monolayer upon 980 nm laser excitation. **b,** Upconversion enhancement factors as a function of pumping power for Er@Y and Tm@Eu samples, respectively. Error bars represent ± 1 s.d. with $n = 3$ each.

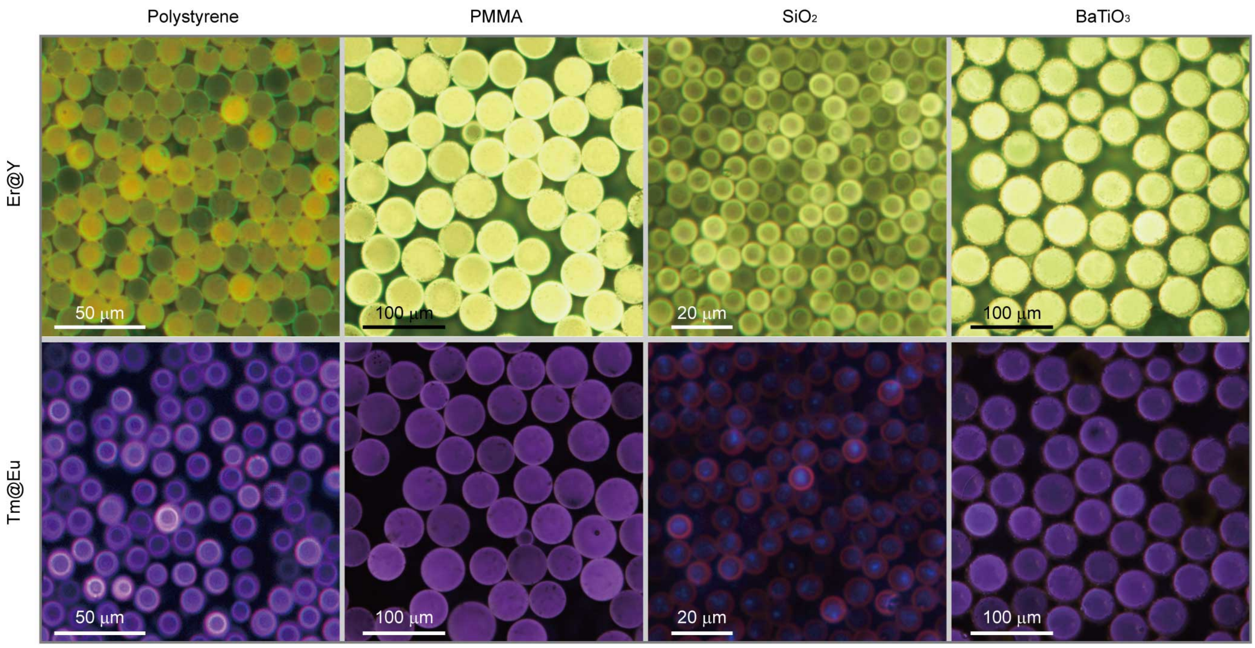

Supplementary Figure 10. Demonstration of the capability of upconversion luminescence enhancement using various organic and inorganic dielectric microbeads. The polymeric (polystyrene, PMMA) and inorganic (SiO_2, $BaTiO_3$) microbeads under study exhibit significant enhancement in the upconversion luminescence of Er@Y and Tm@Eu samples. Refractive indices at 980 nm of polystyrene, PMMA, SiO_2, $BaTiO_3$ are 1.57, 1.48, 1.45, and 2.1, respectively.

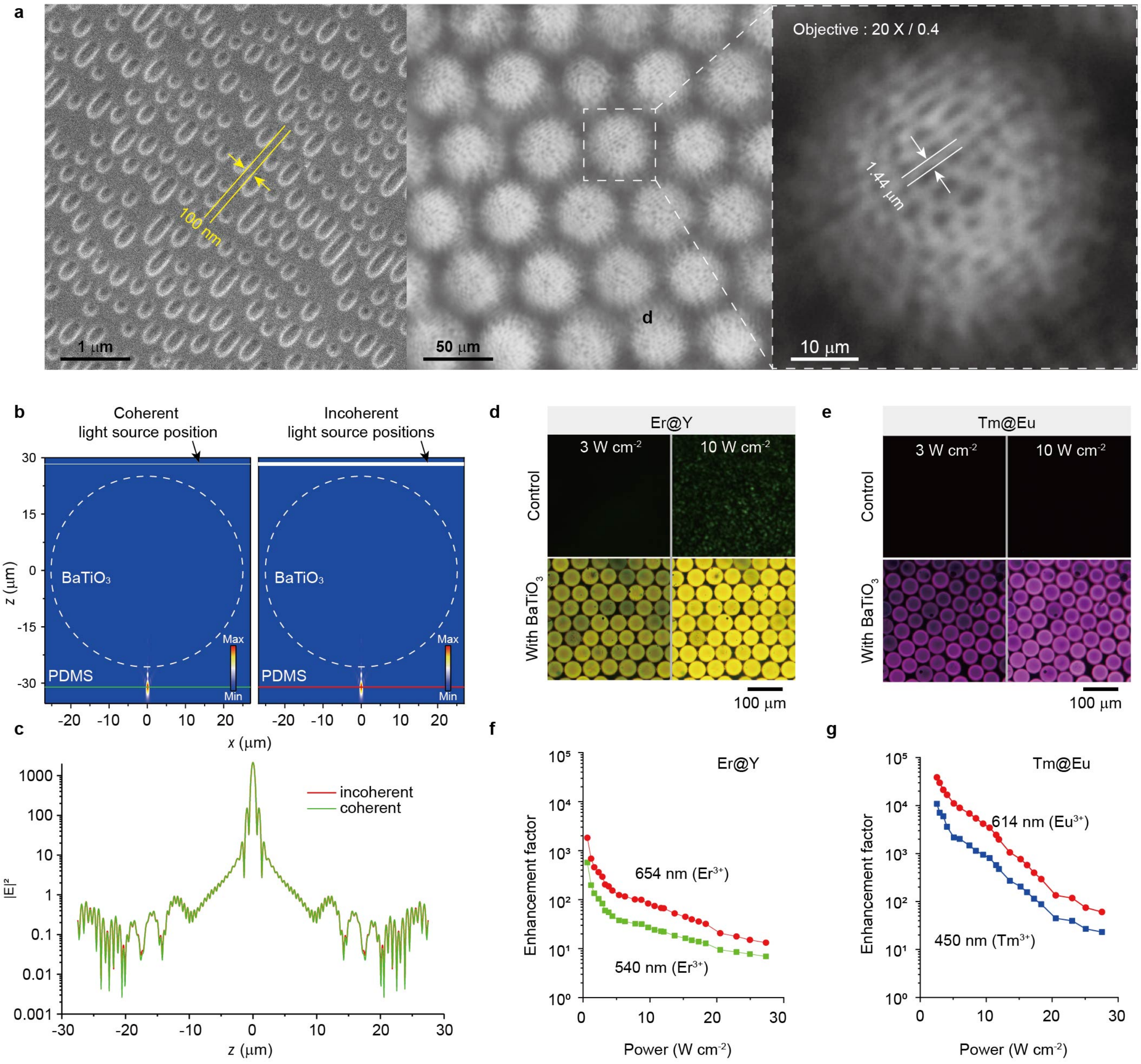

Supplementary Figure 11. Upconversion luminescence amplification with a large-area and portable $BaTiO_3$/PDMS superlensing film. a, Super-resolution imaging of a blue-ray disk with the $BaTiO_3$/PDMS film using a conventional fluorescence microscope. **b,** FDTD simulation revealing the light condensing ability of the $BaTiO_3$/PDMS superlensing system. For modeling with an incoherent light source, the simulation result is obtained by superimposing 11 models with light sources at different positions along z-axis. **c,** Comparison of the light distribution of the nanojet illuminated by coherent and incoherent light. **d, e,** Upconversion luminescence images of the $BaTiO_3$/PDMS film, recorded at two different pumping powers for Er@Y and Tm@Eu samples with and without the microbead monolayer coverage. **f, g,** Upconversion enhancement factors measured as a function of pumping power for Er@Y and Tm@Eu samples.

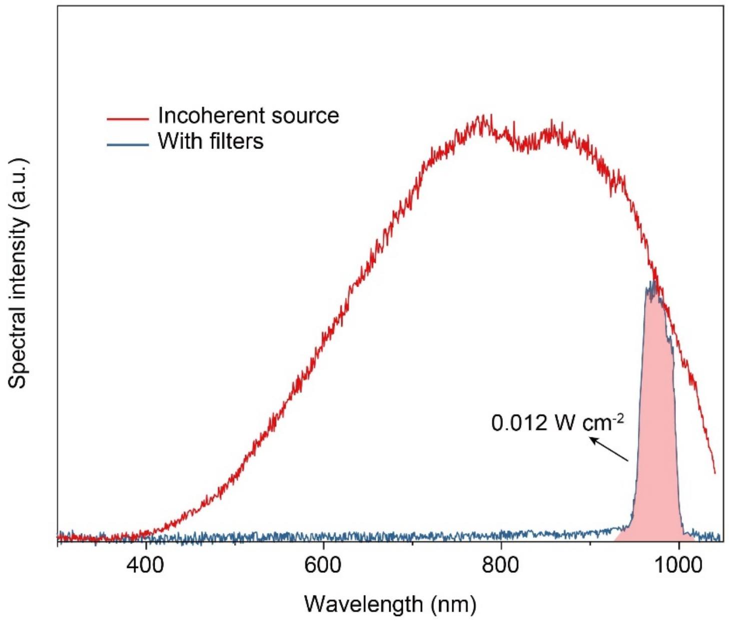

Supplementary Figure 12. The spectral intensity distribution of tungsten incoherent light source. Note that a filter set of a 950-nm long pass and a 1000-nm short pass was used for NIR light extraction.

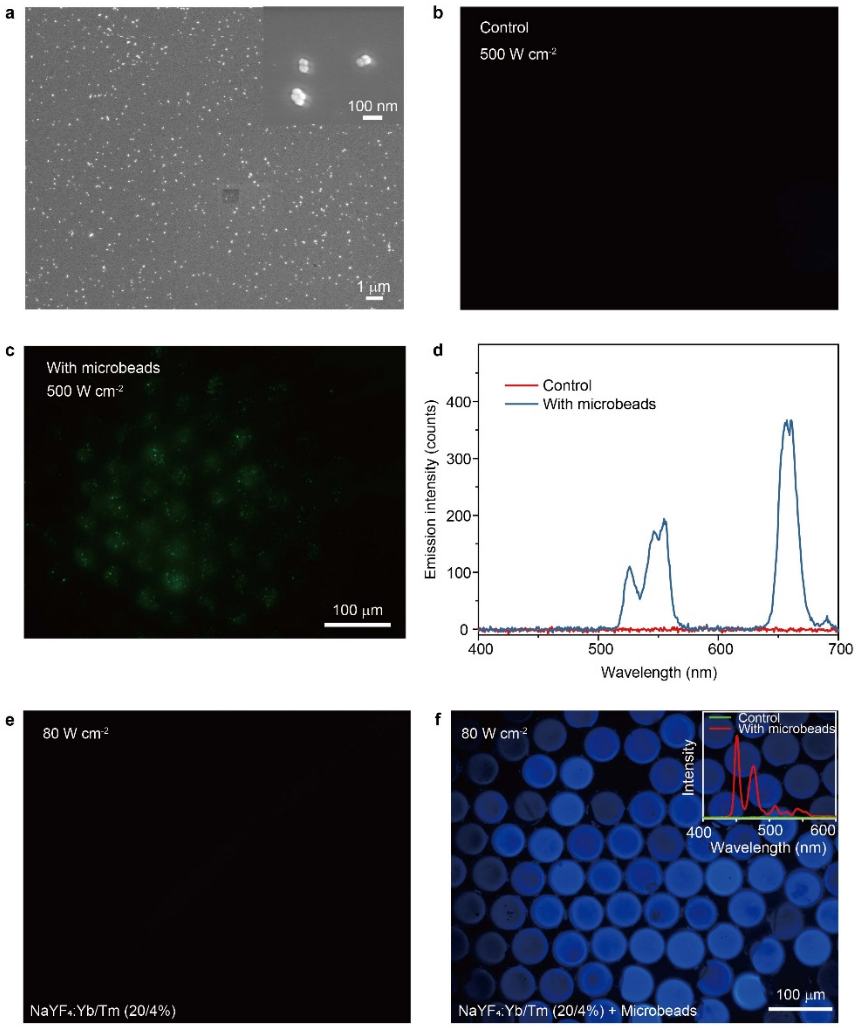

Supplementary Figure 13. Demonstration of superior upconversion enhancement of the $BaTiO_3$/PDMS composite film for single UCNP and heavily Tm^{3+}-doped UCNPs. a, SEM image of the $NaYF_4$:Yb/Er@$NaYF_4$ core-shell UCNPs under study. **b**, **c,** The corresponding wide-field optical microscopic images of the core-shell UCNPs, recorded without and with the coverage of the dielectric superlensing composite film. **d,** The corresponding upconversion emission spectra. **e**, **f,** Wide-field optical microscopic images of $NaYF_4$:Yb/Tm (20/4%) core UCNPs without (**e**) and with (**f**) the coverage of the $BaTiO_3$/PDMS film. (Inset in **f**) The corresponding emission spectra of **e** and **f**.

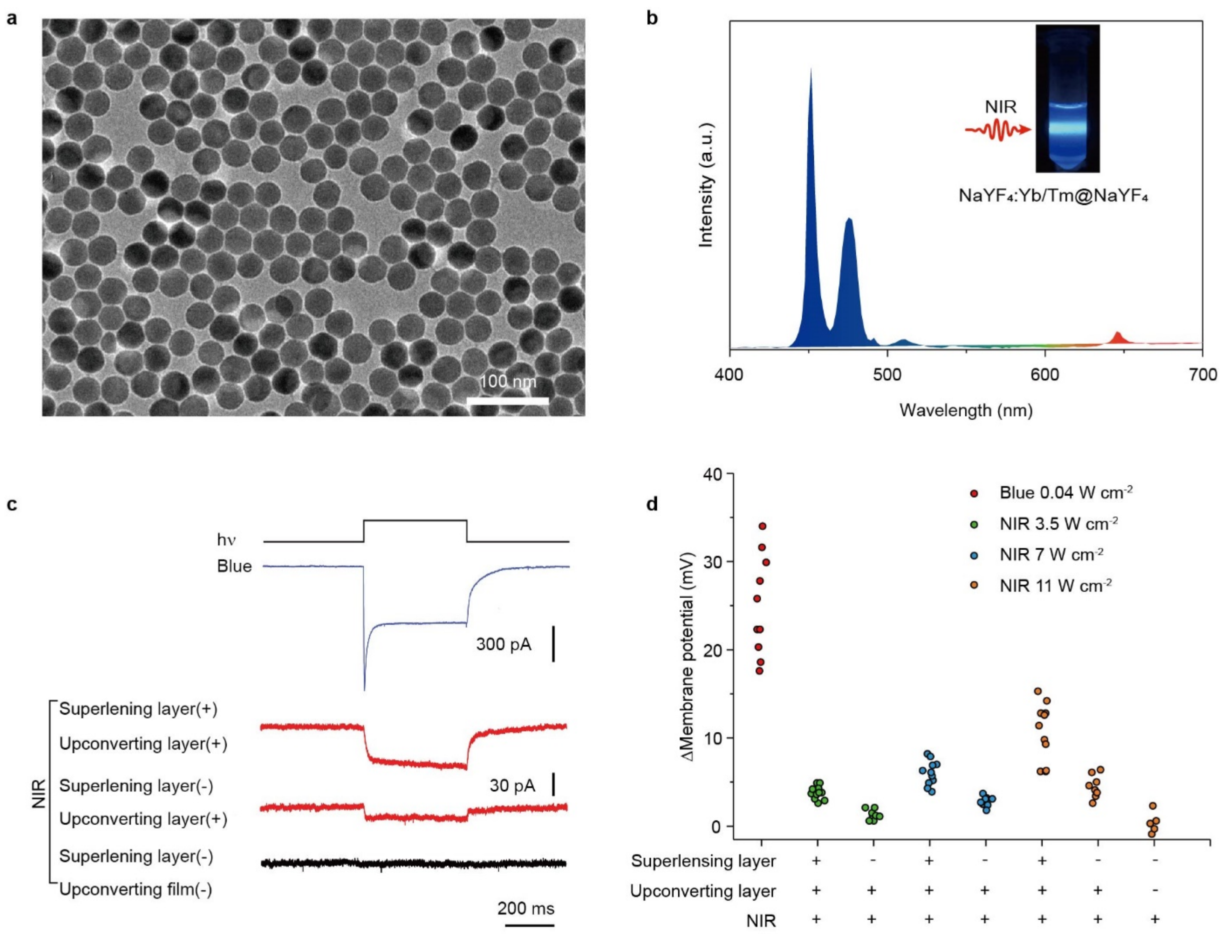

Supplementary Figure 14. NIR-based optogenetics through the use of UCNPs and $BaTiO_3$ microbeads. a, TEM image of the as-prepared $NaYF_4$:Yb/Tm@$NaYF_4$ nanocrystals. **b,** Upconversion emission spectrum of the $NaYF_4$:Yb/Tm@$NaYF_4$ nanocrystals. The inserted image is the corresponding photography of the $NaYF_4$:Yb/Tm@$NaYF_4$ colloidal solution under 980 nm laser excitation. **c,** Voltage-clamp traces of a brain slice containing MCH neurons in response to 500 ms blue LED light or 980 nm light stimulation. Note that MCH neurons were voltage clamped at -60 mV. **d,** The variation in membrane potential induced by blue LED light or 980 nm NIR light at various intensities (for 3.5 and 7 W cm^{-2}, $*p$ = 2.65 x 10^{-5}; for 11 W cm^{-2}, $*p$ = 1.59 x 10^{-4}). Source data are provided as a Source Data file.
