# Supplementary material for: Upconversion amplification through dielectric superlensing modulation
Source: Nat Commun. 2019 Mar 27;10:1391. doi: 10.1038/s41467-019-09345-0 (PMC6437158; doi:10.1038/s41467-019-09345-0)
Supplement: Supplementary file 3 — Description of Additional Supplementary Files [file 41467_2019_9345_MOESM3_ESM.pdf]

**Description of Additional Supplementary Files**

File Name: Supplementary Movie 1
Description: The movie shows the upconversion fluorescence imaging of upconversion nanoparticle-embedded PDMS films with and without the coverage of PEGDA polymeric microbeads.

File Name: Supplementary Movie 2
Description: The movie shows the comparison of blue upconversion emission intensities at varied excitation intensities between upconverting PDMS films with and without the coverage of a dielectric microbead layer.
